# Nanoencapsulated essential oils embedded in ice improve the quality and shelf life of fresh whole seabream stored on ice

**DOI:** 10.1016/j.heliyon.2019.e01804

**Published:** 2019-06-18

**Authors:** Laura Navarro-Segura, María Ros-Chumillas, Amanda E. López-Cánovas, Alfonsa García-Ayala, Antonio López-Gómez

**Affiliations:** a Department of Food Engineering and Agricultural Equipment, Universidad Politécnica de Cartagena, Paseo Alfonso XIII, 48, E-30203, Cartagena, Spain; b Instituto de Biotecnología Vegetal, Campus de Excelencia Internacional Regional "Campus Mare Nostrum", Universidad Politécnica de Cartagena, Campus Muralla del Mar, E-30202, Cartagena, Spain; c Department of Cell Biology and Histology, Faculty of Biology, Universidad de Murcia, Campus de Espinardo, E-30100, Murcia, Spain

**Keywords:** Food safety, Food technology, Food microbiology, Nanotechnology, Food preservation, Ice-storage, β-Cyclodextrin, Freshness quality, Microbial quality, Sensory quality

## Abstract

Ice containing essential oils (EOs) nanoencapsulated in β-cyclodextrins (β-CD) (named as EOs+β-CD ice) was used for stunning/slaughtering by hypothermia in ice slurry, and for ice storage of gilthead seabream. Clove essential oil (CEO) was used at fish stunning/slaughtering, while ice storage of whole fish was performed using a combination of carvacrol, bergamot and grapefruit EOs (CBG). Inclusion complexes CBG+β-CD were characterized, and antimicrobial effect was also evaluated. The kneading method used to form inclusion complexes with CBG showed a good complexation efficiency. Microbial, physical-chemical and sensory analyses were carried out to assess the quality changes of fresh whole seabream during ice storage at 2 °C for 17 days. Results (microbial, chemical and sensorial) indicated that seabream stunning/slaughtering and storage using EOs+β-CD ice (in low doses of 15 mg/kg ice for stunning, and 50 mg/kg ice for ice storage) improved the quality of fresh fish and extended the shelf-life up to 4 days.

## 1 Introduction

Seabream (*Sparus aurata*) is one of the main fish species farmed in numerous Mediterranean countries, due to their appreciate characteristics, reaching a total production of 207.167 tons in 2017 (APROMAR, 2018). However, fresh seabream is highly susceptible to spoilage during ice storage, due to enzymatic and chemical reactions-usually responsible for the initial loss of freshness-, and microbial activity that is responsible for the obvious spoilage and its short shelf-life of 14–15 days (Carrascosa et al., 2015; Kriton et al., 2018).

Conservation on ice is a method widely used for fresh fish due to its cooling efficiency, temperature control, moisture retention, safety, ease of use, availability, and cost-effectiveness (Tavakoli et al., 2018). Thus, the enhancement of this method for increasing the shelf-life of fresh fish could have a great impact in the fish sector. This is the reason why methods of icing using various antimicrobial and antioxidants, as essential oils (EOs) have been reported (Tavakoli et al., 2018). In fact, EOs are an alternative to chemical preservatives because consumers have expressed a desire to reduce the use of synthetic chemicals in foods (Hassoun and Çoban, 2017; Gokoglu, 2018).

In recent years, only a few researches have evaluated the combined effect of icing and essential oils applied directly in fresh fish. For example, Özyurt et al. (2012) investigated the effect of icing with rosemary extract on the quality of sardines and found that this method improved the quality and safety of sardine. Tavakoli et al. (2018) also found a shelf-life enhancement of chilled whole rainbow trout (*Oncorhynchus mykiss*) treated with Reshgak EOs ice coverage, reaching a shelf-life of 16 days compared to 12 days reached by traditional icing.

In these studies, the EOs doses used are relatively high. For example, Tavakoli et al. (2018) used a dose of 1500 mg/kg ice of Reshgak EOs. Furthermore, EOs usually have a strong flavour that can affect negatively the sensory properties of fish (Hassoun and Çoban, 2017; Atarés and Chiralt, 2016). In order to avoid these drawbacks, as EOs losses by high volatility and undesirable sensory effects, cyclodextrins (CDs) could be used to encapsulating EOs (Fenyvesi et al., 2016).

CDs are cyclic oligomers of α-D-glucopyranose, of which β-cyclodextrins (β-CD) are the most used in food technology. The β-CD molecule exhibits the shape of a truncated hollow cone, which interior cavity is hydrophobic, and the exterior is hydrophilic. These characteristics allow β-CD be a good tool for increasing water solubility and protecting EOs against oxidation and evaporation. Hence, β-CD have been used in food technology for molecular encapsulation of EOs –forming an inclusion complex named as EOs+β-CD in this work–, which have been recognised as GRAS (Fenyvesi et al., 2016). Kneading method gives an excellent yield of inclusion of EOs in β-CD molecule (Kamikura et al., 2014). Notwithstanding the above, the characterization of inclusion complex has been studied usually only for one type of EOs (Kamikura et al., 2014; Menezes et al., 2016; Piletti et al., 2017), and not for a combination of different EOs.

The major compounds of bergamot (*Citrus bergamia Risso*) EOs, which have a fresh and elegant aroma, are limonene, linalyl acetate, linalool, γ-terpinene, and β-pinene. Grapefruit EOs are a mixture of volatile compounds like terpenes and oxygenated derivatives such as aldehydes (citral), alcohols and esters, their main component being D-limonene. Bergamot and grapefruit extracts have been studied to inhibit the oxidation of fish lipid with successful results, and were the most preferred citrus in terms of sensory analyses (Yerlikaya et al., 2016).

By other hand, according to research literature, EOs from oregano are one of the most used antimicrobial and antioxidant agents in fish and seafood products (Hassoun and Çoban, 2017). The major compounds of oregano EOs (OEOs) are carvacrol and thymol (phenols) which have antioxidant and antimicrobial activity (Juliano et al., 2000). Carvacrol is reported to be the main compound responsible for the antimicrobial and antioxidant activity of OEOs (Rodriguez-Garcia et al., 2016).

As far as our knowledge is concerned, the use of nanoencapsulated EOs embedded in ice (EOs+β-CD ice) for storage of fresh whole fish in ice has not been studied. Thus, this work characterizes the quality consistency of the newly prepared EOs+β-CD and also studies the use of EOs+β-CD ice for ice-storage of fresh seabream to improve its freshness quality and shelf-life, and in combination or not with the use of clove EOs (CEO) nanoencapsulated in β-CD, and embedded in ice, for stunning and slaughtering of farmed seabream and for decreasing its stress, as described in López-Cánovas et al. (2019).

## 2 Materials and method

### 2.1 Essential oils and ice preparation

Bergamot, carvacrol and grapefruit essential oils were obtained from Lluch Essence (Spain). β-CDs was provided by Roquette (France). The EOs+β-CD inclusion complex powder was prepared using 1:1 molar ratio (1g of CBG and 7.6 g of β-CD) following the kneading method (Marques, 2010), and the crushed ice with EOs+β-CD embedded were manufactured by adding EOs+β-CD powder in potable water before entering in the ice machine (Vogt Ice, USA). The different EOs+β-CD concentrations are expressed as mg of EOs per kg of ice.

In previous studies, the most effective dose of EOs (as combination of EOs of bergamot and grapefruit, and carvacrol, -in a ratio of 3:1:1-, and forming inclusion complex with β-CD, named CBG+β-CD) for the conservation of fish on ice was selected. Doses were evaluated from 25 to 100 mg EOs/kg of ice, and the dose of 50 mg EOs/kg of ice was finally selected because it was adequately effective in keeping the freshness of the fish on ice.

### 2.2 Characterization of the inclusion complex

#### 2.2.1 Differential scanning calorimetry (DSC)

The Differential Scanning Calorimetry (DSC) technique was utilized to analyze the thermal behavior of the EOs+β-CD inclusion complexes. The analyses were performed using a differential scanning calorimeter DSC 822e (Mettler-Toledo GmbH, Schwerzenbach, Switzerland). Ten mg of each sample were placed in 100 μL sealed aluminum crucibles whose lips were perforated with a 50 μm diameter pin. Thermograms were obtained at temperatures of 25 °C–400 °C, with a heating rate of 10 °C min^−1^ and under a nitrogen atmosphere with a flow rate of 50 mL min^−1^. Thermograms were obtained for pure carvacrol essential oil, pure mix of carvacrol, bergamot and grapefruit (3:1:1; v/v), pure β-cyclodextrine and the EOs+β-CD complexes.

#### 2.2.2 Entrapment efficiency (EE%)

The amount of EOs entrapped in the inclusion complex was determined by UV-Vis spectroscopy (TECAN Infinitive M200, Männedorf, Switzerland) at 254 nm. 5 mg of sample was dissolved in 5 mL of acetonitrile and left for 48 h after being well mixed to allow enough time for all entrapped active compound to be in solution. After this procedure, the solutions were centrifuged at 3200 rpm for 15 min to remove any β-CD from the solution, leaving only the active compound (carvacrol, bergamot and grapefruit). A standard curve of carvacrol was prepared with concentration ranging of 2.5–30 μg/mL, under the same conditions. The EE% was calculated as follow:

$$\text{EE}=\frac{\text{amount}\;\text{of}\;\text{EOs}\;\text{entrapped}}{\text{intial}\;\text{EOs}\;\text{amount}}x100$$

where amount of EOs entrapped was the difference between total EOs and surface-adsorbed EOs in the inclusion complex (Santos et al., 2015; Kamikura et al., 2014; Menezes et al., 2016).

#### 2.2.3 Fourier transform infrared spectroscopy (FTIR)

FITR used 10 mg of each samples, pure mix of carvacrol, bergamot and grapefruit (3:1:1; v/v) and the EOs+β-CD complexes. Attenuated total reflectance–Fourier transform infrared spectra were obtained by means of a Nicolet 5700 spectrometer (Thermo Inc., Waltham, MA, USA) using a single-bounce diamond attenuated total reflection accessory (Smart Orbit, Thermo Inc.) at room temperature (25 °C).

Spectral data were treated in the range of 3600–2400 cm^−1^ with OMNIC 8.0 software (Thermo Fisher Scientific Inc.), although the complete spectrum was collected in the 4000–400 cm^−1^ range.

#### 2.2.4 Scanning electron microscopy (SEM)

The morphology of β-cyclodextrins and the EOs+β-CD complexes was analyzed using a Scanning Electron Microscope (Model S-3500N Hitachi). The samples were coated with a thin film of platinum and analyzed at different magnifications: 1000x and 2000x.

### 2.3 Raw fish, stunning/slaughtering and ice-storage

The seabream fish (650 ± 50 g body mass, bm) were obtained from a fish farm of the company Servicios Atuneros del Mediterráneo SL (San Pedro del Pinatar, Murcia, Spain) located in South-eastern Spain. The stunning/slaughtering of farmed fish was carried out by hypothermia in crushed ice slurry (with a ratio of 1:1:1, sea water:ice:fish, w/w/w). It was used normal crushed ice (without EOs+β-CD), and crushed ice including CEO inclusion complex in β-CD (CEO+β-CD) in two concentrations: 10 and 15 mg CEO/kg ice. The stunning/slaughtering of seabream was conducted as described in López-Cánovas et al. (2019). After 2 hours of post-mortem, all the fish was placed in polystyrene boxes, and covered with crushed ice for ice-storage (in a ratio ice:fish of 1:3, w/w). The crushed ice for fish conservation was of two kinds: normal crushed ice (control), and antimicrobial crushed ice (containing CBG+β-CD in a concentration of 50 mg of EOs per kg of ice).

The different treatments studied in this work are the followings:

- Current slaughtering and ice storage using normal crushed ice (control).
- Current slaughtering with normal ice and ice storage using CBG+β-CD ice.
- Stunning/slaughtering with 10 mg CEO+β-CD ice slurry, and storage with normal crushed ice.
- Stunning/slaughtering with 10 mg CEO+β-CD ice slurry, and storage with CBG+β-CD crushed ice.
- Stunning/slaughtering with 15 mg CEO+β-CD ice slurry, and storage with normal ice.
- Stunning/slaughtering with 15 mg CEO+β-CD ice slurry, and storage with CBG+β-CD crushed ice.

The polystyrene boxes were provided with holes for water drainage, and were transported to the pilot plant of the Universidad Politécnica of Cartagena to be stored in a cold room at 2 ± 0.5 °C for 17 days. Melting ice was replaced daily with a fresh quantity and excess water was drained.

Fish samples (4 units) were taken on day 1, 7, 11, 15 and 17 from ice storage to perform the different analyses. One fish unit was for physicochemical and microbiological analyses, and the other three for the sensory analysis.

The experiments described were approved by the General Directorate of Agriculture, Livestock, Fisheries and Aquaculture (Department of Water, Agriculture and Environment) of the Autonomous Community of the Region of Murcia (Spain; approval number A13160508). These experiments were conducted according to established animal welfare guidelines, and this study complies with all regulations.

### 2.4 Microbiological analysis

For microbiological analyses, 25 g of fish muscle sample from dorsal region was diluted with 225 mL sterile buffered peptone water (Scharlau Chemie) in a sterile stomacher bag (Model 400 Bags 6141, London, UK) and homogenized for 2 min using a masticator (Colwort Stomacher 400 Lab, Seward Medical, London, UK). Serial dilutions of each suspension were carried out in sterile peptone water (Scharlau Chemie), and analysed for microbial counts.

Appropriate aliquots (0.1 or 1 mL) were spread on agar plates. Plate Count Agar (Scharlau Chemie) was used for enumeration of mesophilic and psychrotrophic aerobic bacteria, and plates were incubated for 48 h at 30 °C and 7 days at 4 °C respectively. *Pseudomonas sp*. were determined on Cetrimide Agar (Scharlau Chemie) after incubation at 37 °C for 48 h. Lactic acid bacteria were counted on Man Rogosa and sharpe Agar (Scharlau Chemie) incubated at 31 °C for 48 h. *Enterobacteriaceae spp*. were counted on violet red bile glucose agar (Scharlau Chemie) incubated at 37 °C for 24 h. All plates were performed in duplicate and the results were expressed as logarithms of the number of colony-forming units per gram (log CFU/g).

### 2.5 Physicochemical analyses

The pH was measured with a pH meter (pH-metro Basic 20, Crison). Fish muscle (5 g) was homogenized thoroughly with 10 mL of distilled water and the homogenate was used for pH determination. Trimethylamin nitrogen **(**TMA-N) was determined using the Picric Acid Method (AOAC, 1998) and measured by spectrophotometer at 410 nm. TMA-N content was expressed as mg N/100 g fish muscle. The Water-holding capacity (WHC) analysis was based on the method described by Grau and Hamm (1953), and WHC was expressed in percentage.

### 2.6 Colour measurements

Colour was measured on muscle from dorsal region surface by L*, a* and b* system CIE (1986), using a Minolta Chroma Meter CR400 (Minolta, Osaka, Japan). Each value was the average of three determinations per samples. In the CIE Lab system, L* denotes lightness on a 0–100 scale from black to white; a*, (+) red or (-) green; and b*, (+) yellow or (-) blue (Schubring, 2002).

### 2.7 Sensory evaluation

The sensory attributes of fish were evaluated by a panel of seven experienced judges and for each day of sampling. The assessment of fish was conducted using the Tasmanian Food Research unit (TRFU) system modified by Cakli et al. (2007) and Erikson et al. (2019), and developed as quality index method (QIM). The panelists evaluated the parameters for 3 sea breams from each condition. On the day of each analysis, and for each parameter, the average score of panellists was obtained. The sea bream scheme ranges from 0 to 40 demerit points. Thus, panelists evaluated the following QI attributes of sea bream: skin appearance (0: bright, iridescent pigmentation, 1: slightly bright, 2: rather dull, becoming discolored, 3: green, yellowish, mainly near abdomen 4: pale, dull); belly and operculum (0: grey, silver, 1: grey, yellowish spots, 2: grey, brown spots); skin odor (0: fresh seaweedy, 1: neutral, 2: cucumber, metal, 3: sour, dish cloth, 4: rotten); skin texture/firmness (0: firm, 1: rather soft, 2: very soft, 3:deformed); mouth resistance (0: very, 1: little, 2: without resistance); mouth color (0: pinky, 1: yellowish); eye cornea (0: clear, translucent 1: opaque and/or red, 2: lightly milky, 3: milky gelatinous); eye pupils (0: black, metal shiny, 1: slightly milky, 2: milky, opaque 3: white, matt); eyes shape (0: convex, 1: flat, 2: sunken, 3: deformed); gill color (0: bright red, 1: pale red, pink, 2: light brown, 3: brown, grey); gill odor (0: fresh, seaweed, neutral, 1: metal, grass, 2: sour, moldy, dish cloth, 3: rotten); gill mucus (0: absent, 1: transparent, 2: milky, clotted, 3: brown, clotted); anal area: mucus (0: absent, 1: lightly, 2: present); anal area: aspect (0: shut, 1: lightly open, 2:open). Accordingly, the modified total QI score ranged from 0 (very fresh fish) to 40 (spoiled fish).

Furthermore, sensory evaluation by panellists was also realised for cooked seabream muscle. Fish muscle sample (25 g) from dorsal region was cooked individually in a microwave oven at full power (850 W) for 2 min, and then presented to panellists. Sensory evaluation was conducted in individual booths under controlled conditions of light, temperature and relative humidity. Panellists were asked to score odour, taste and texture of fish using a 0–10 descriptive hedonic scale (Papadopoulos et al., 2003).

### 2.8 Statistical analysis

Results are reported as mean values ± standard error. For data analysis, ANOVA and standard derivation were used. Significant differences were defined as *p*˃0.05. Data were subjected to analysis of variance (ANOVA), followed by Tukey HSD multiple range tests to evaluate the significant difference (*p*˂0.05) among the different treatments. A regression analysis was used to determine the shelf-life of seabream. The data were fitted by a linear model, Y = a+bX, where Y represents mesophilic aerobic count and X represents the time of ice storage.

## 3 Results and discussion

### 3.1 Characterization of inclusion complexes

#### 3.1.1 Differential scanning calorimetry (DSC)

**Fig. 1:**
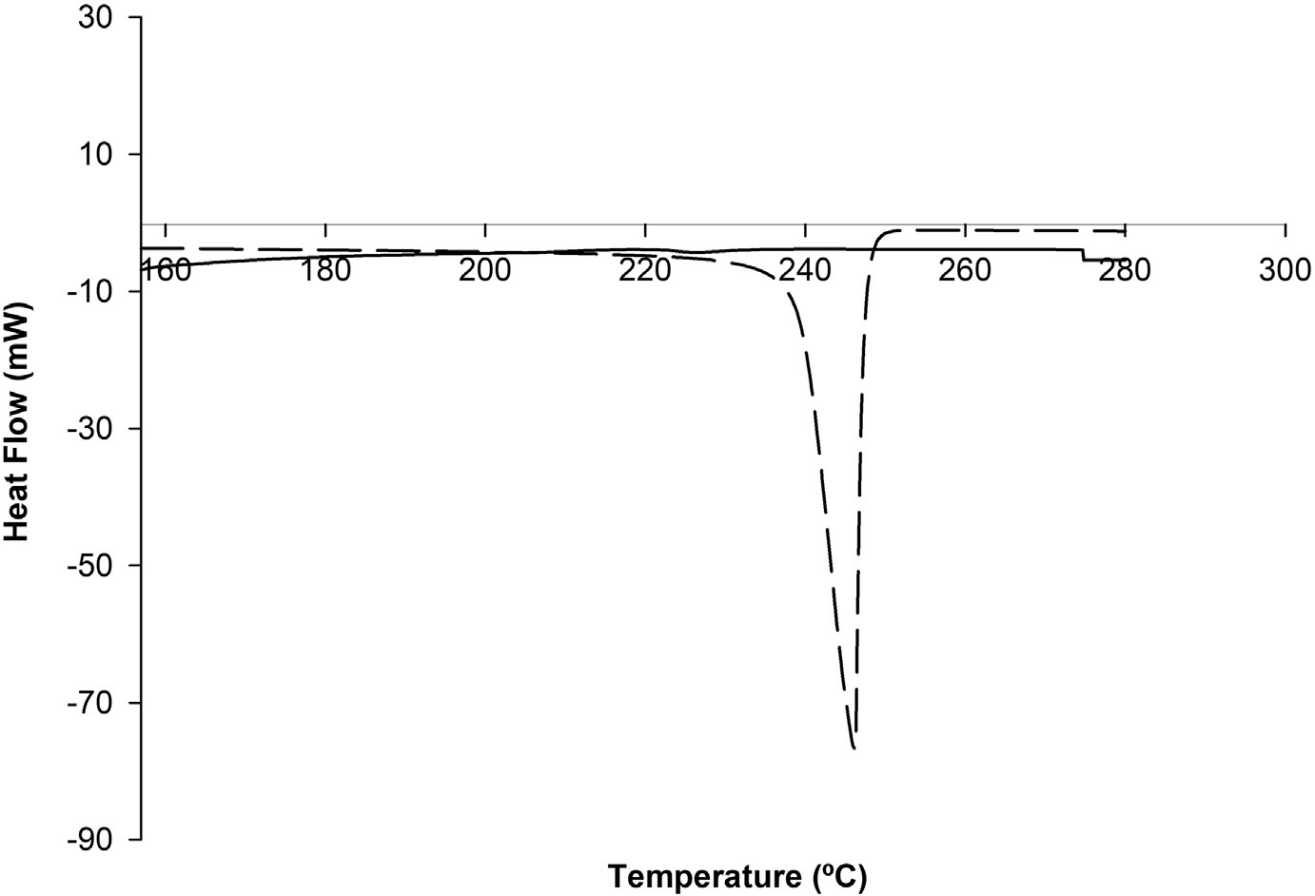
Differential scanning calorimetry (DSC) thermograms of carvacrol (long dash) and β-CD+carvacrol inclusion complexes (solid line).

**Fig. 2:**
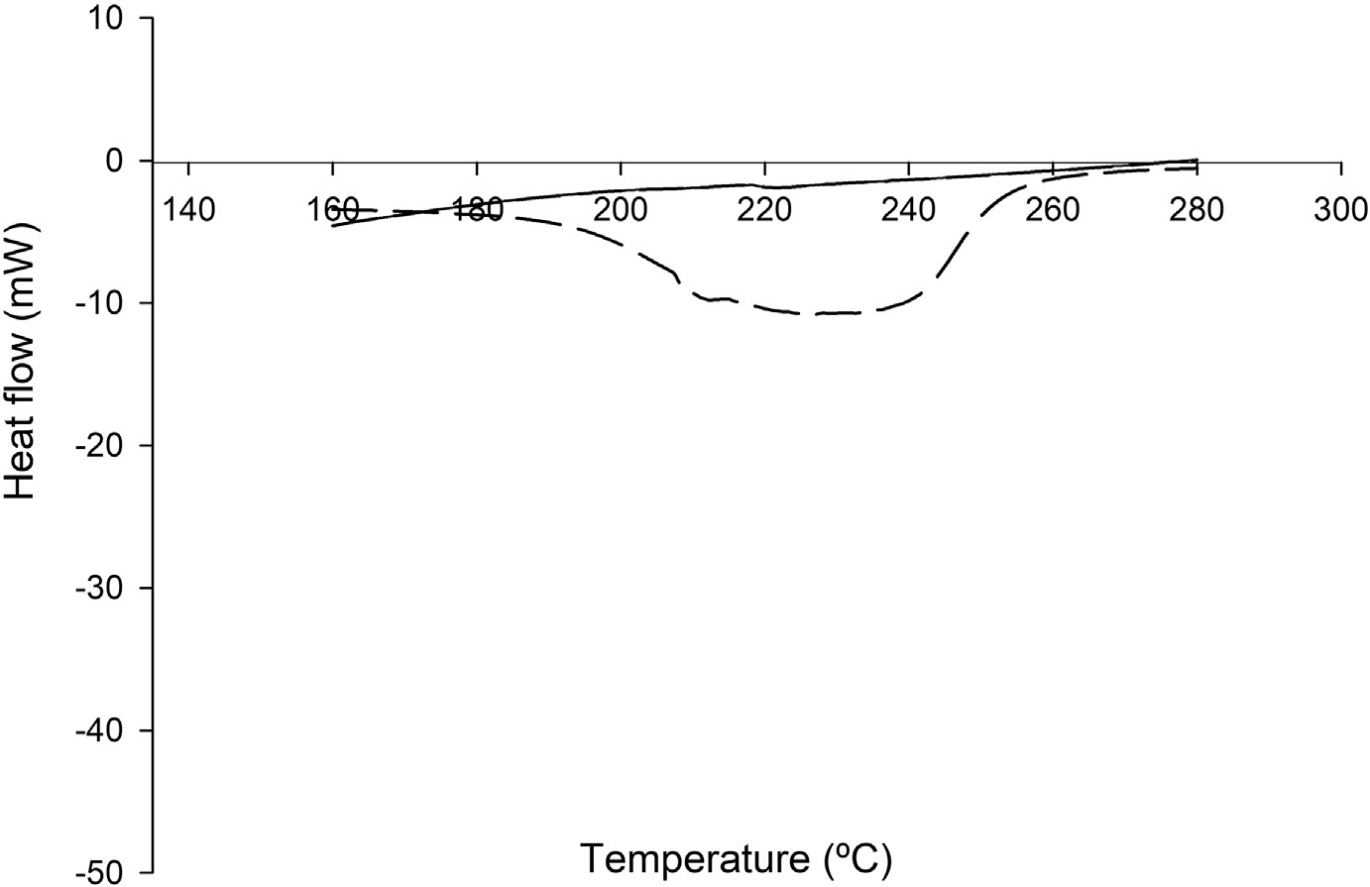
Differential scanning calorimetry (DSC) thermograms of mix of carvacrol, bergamot and grapefruit essential oils (3:1:1) (long dash) and β-CD+carvacrol inclusion complexes (solid line).

Fig. 1 shows the thermograms obtained by DSC for carvacrol alone and β-CD-carvacrol inclusion complexes. The DSC curve of carvacrol shows a sharp endothermic peak at 241.17 °C, corresponding to its boiling point, whereas the DSC curve of β-CD+carvacrol did not show the carvacrol peak, indicating the molecular encapsulation of the carvacrol inside the β-CD cavity. Other authors (Kamikura et al., 2014; Santos et al., 2015; Piletti et al., 2017**)** found similar results. The DSC curve of mix of carvacrol, bergamot and grapefruit (3:1:1) and β-CD+carvacrol, bergamot and grapefruit (β-CD+CBG) inclusion complexes are shown in Fig. 2. The DSC curve of mix of carvacrol, bergamot and grapefruit show a broad endothermic peak at 233.62 °C, corresponding to different boiling point in mix essential oils. As in the other inclusion complexes, the DSC curve of β-CD+carvacrol, bergamot and grapefruit inclusion complexes showed disappearance of the mix essential oils. Hence, the formation of inclusion complexes was confirmed by DSC analysis because the elimination of the peaks is an indirect proof that an inclusion complex has been formed (Kamikura et al., 2014; Santos et al., 2015**). T**hermograms for β-CDs structure were relatively stable between temperatures of 100 °C and 250 °C as shown in Figs. 1 and 2. Piletti et al. (2017) found similar results.

#### 3.1.2 Entrapment efficiency (EE)

EE is used to determine the amount of EOs entrapped in the inclusion complex. The percentage of complexation of EOs in the cavity of β-CD was 68.16%. Other authors found similar results. Menezes et al. (2016) found values of 71.68% in β-CD inclusion complex with carvacrol prepared with slurry complexation method and 34.30% in β-CD inclusion complex with carvacrol prepared with paste complexation method. Kamikura et al. (2014) showed that kneaded and freeze-dried methods used to obtain inclusion complexes entrapped carvacrol very effectively. They found EE values of 78.09 and 83.74% for both inclusion complexes methods. Hence, kneading method entrapped mix of CBG effectively.

#### 3.1.3 Fourier transform infrared spectroscopy (FTIR)

**Fig. 3:**
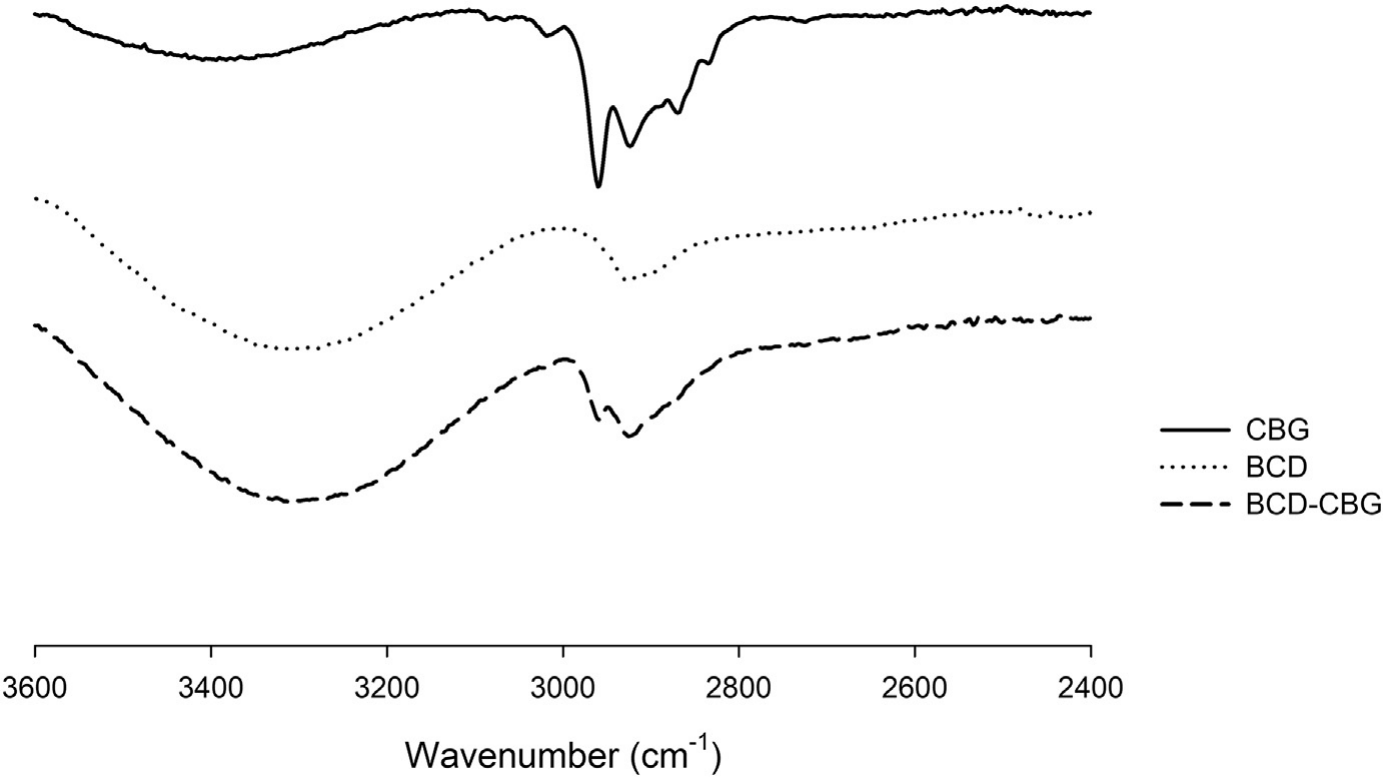
FTIR spectra of CBG, β-CD and β-CD+CBG inclusion complexes obtained in the spectral range of 3600–2400 cm^−1^.

**Fig. 4:**
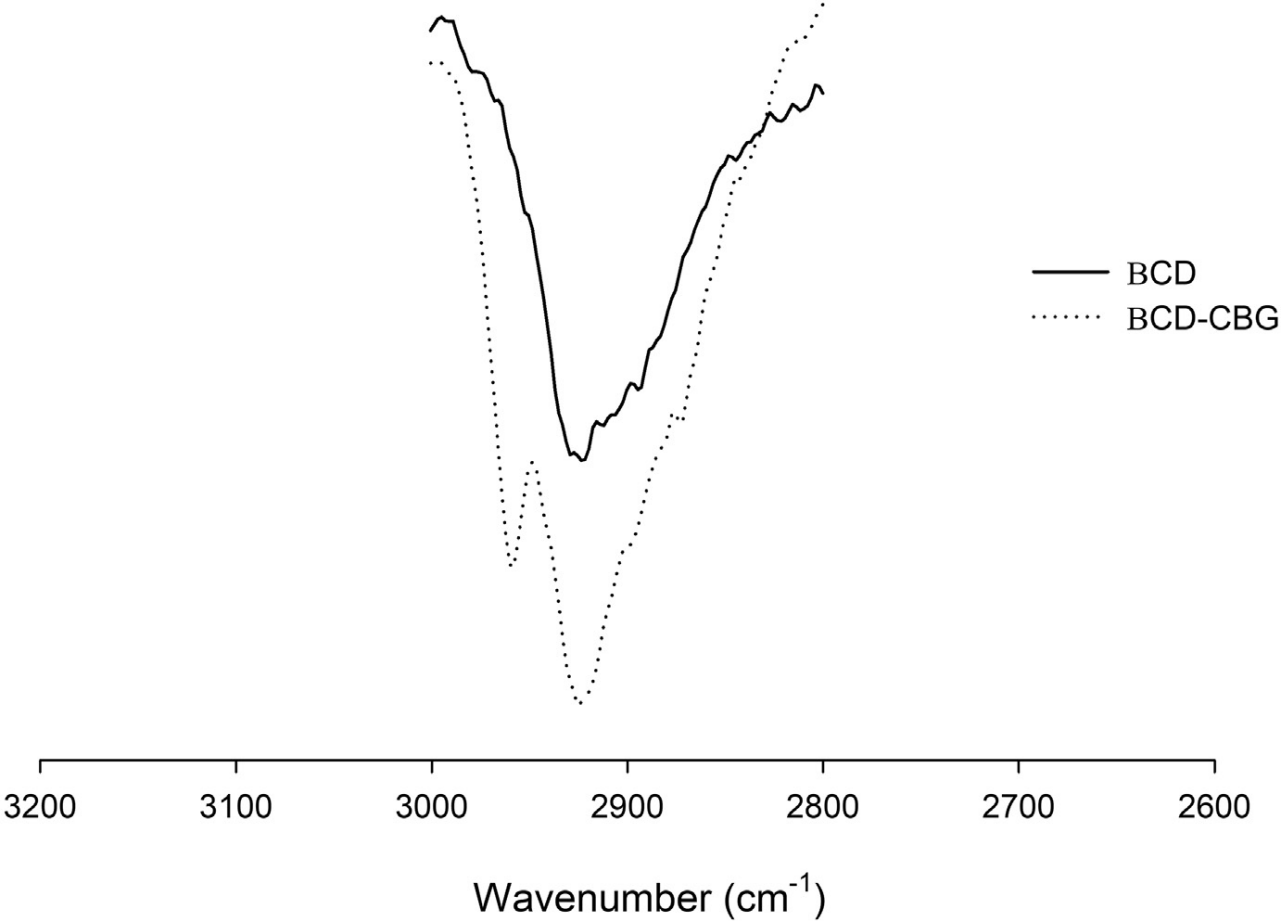
FTIR spectra of β-CD and β-CD+CBG inclusion complexes obtained in the spectral range of 3200–2600 cm^−1^.

The formation of β-CD-CBG inclusion complexes was evaluated by Fourier transform infrared spectroscopy (FTIR). Fig. 3 shows FTIR spectra obtained for CBG, β-CD and β-CD+CBG inclusion complexes for the wavelength range from 3600 to 2400 cm^−1^. FTIR is used to confirm the presence of specific chemical groups in the inclusion complexes (Piletti et al., 2017). The spectrum obtained for the β-CD+CBG inclusion complexes shows typical representation of a physical interaction between CBG mix and the β-CD molecules (Fig. 4). The FITR spectrum is a simple interaction between CBG mix and β-CD molecules. Piletti et al. (2017) found similar results in FITR spectrum obtained for the eugenol, β-CD molecules and eugenol-β-CD inclusion complexes. FTIR spectrum obtained for the β-CD+CBG shows characteristics absorption band of CBG mix and β-CD molecules.

#### 3.1.4 Scanning electron microscopy (SEM)

**Fig. 5:**
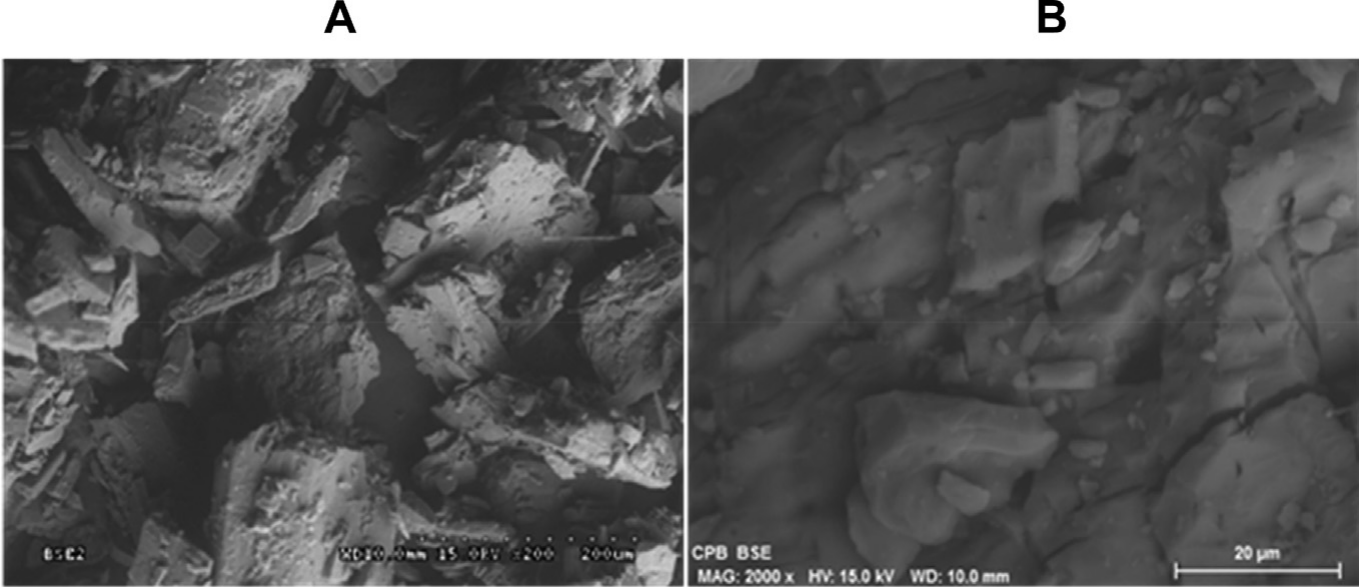
Images obtained by Scanning electron microscopy (SEM) for the β-CD+CBG inclusion complexes (A) with 200x and images obtained for the β-CD+CBG inclusion complexes (B) with 2000x.

In order to assess morphological structure and shape of inclusion complexes, SEM imaging was performed. Images obtained by SEM are shown in Fig. 5. Fig. 5A shows images obtained for β-CD+CBG with 200x. Surface morphology of β-CD appears as irregular crystalline blocky structure with harsh surface and large crystals. Choi et al. (2009), Prabu et al. (2015) and Piletti et al. (2017), reported that presence of large crystals indicates inclusion complex were formed.

### 3.2 Fish microbiology assessment

**Fig. 6:**
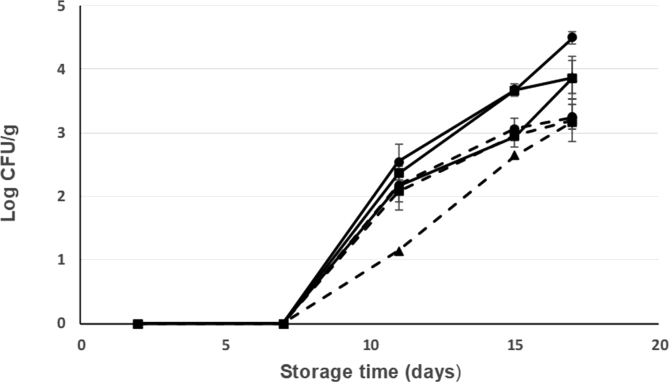
Changes in the counts of *Enterobacteriaceae* bacteria along cold storage (at 2 °C) of fresh seabream with different treatments: Current slaughtering and normal ice storage (control) (---●---); Current slaughtering and storage with CBG+β-CD ice (---●---; dashed line); Stunning/slaughtering with 10 mg CEO+β-CD ice and normal ice storage (---■---); Stunning/slaughtering with 10 mg CEO+β-CD ice and ice storage with CBG+β-CD ice (---■---; dashed line); Stunning/slaughtering with 15 mg CEO+β-CD ice and storage in normal ice (--▲--); Stunning/slaughtering with 15 mg CEO+β-CD ice and storage with CBG+β-CD ice (--▲--; dashed line).

**Fig. 7:**
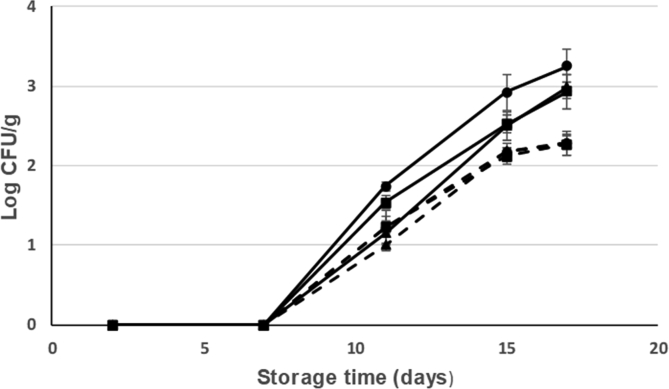
Changes in the counts of *Peudomonas* bacteria along cold storage (at 2 °C) of fresh seabream with different treatments: Current slaughtering and normal ice storage (control) (---●---); Current slaughtering and storage with CBG+β-CD ice (---●---; dashed line); Stunning/slaughtering with 10 mg CEO+β-CD ice and normal ice storage (---■---); Stunning/slaughtering with 10 mg CEO+β-CD ice and ice storage with CBG+β-CD ice (---■---; dashed line); Stunning/slaughtering with 15 mg CEO+β-CD ice and storage in normal ice (--▲--); Stunning/slaughtering with 15 mg CEO+β-CD ice and storage with CBG+β-CD ice (--▲--; dashed line).

**Fig. 8:**
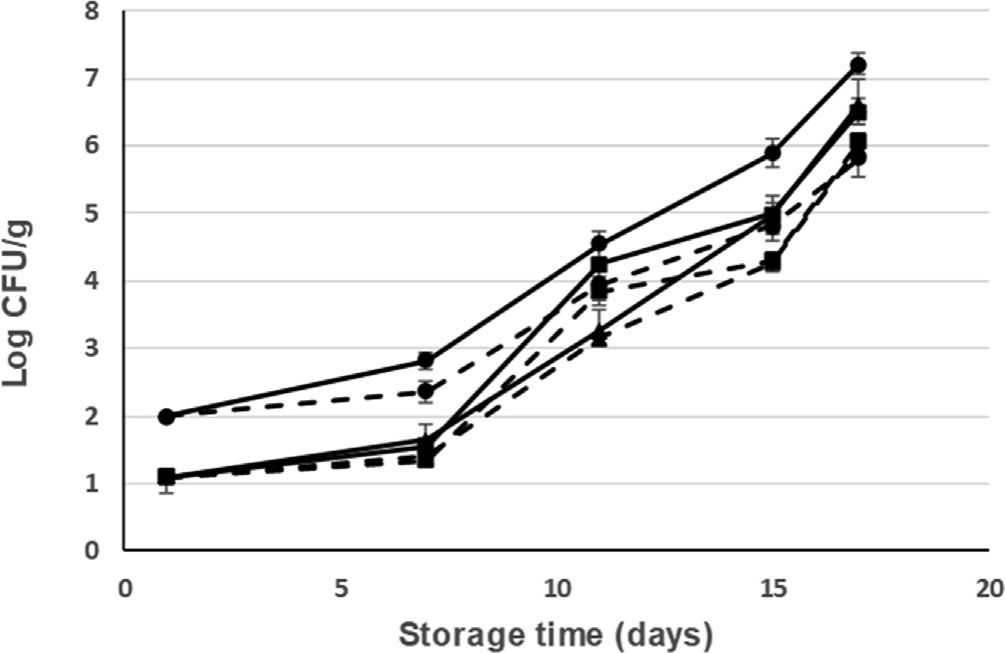
Changes in the counts of mesophilic aerobic bacteria along cold storage (at 2 °C) of fresh seabream with different treatments: Current slaughtering and normal ice storage (control) (---●---); Current slaughtering and storage with CBG+β-CD ice (---●---; dashed line); Stunning/slaughtering with 10 mg CEO+β-CD ice and normal ice storage (---■---); Stunning/slaughtering with 10 mg CEO+β-CD ice and ice storage with CBG+β-CD ice (---■---; dashed line); Stunning/slaughtering with 15 mg CEO+β-CD ice and storage in normal ice (--▲--); Stunning/slaughtering with 15 mg CEO+β-CD ice and storage with CBG+β-CD ice (--▲--; dashed line).

**Fig. 9:**
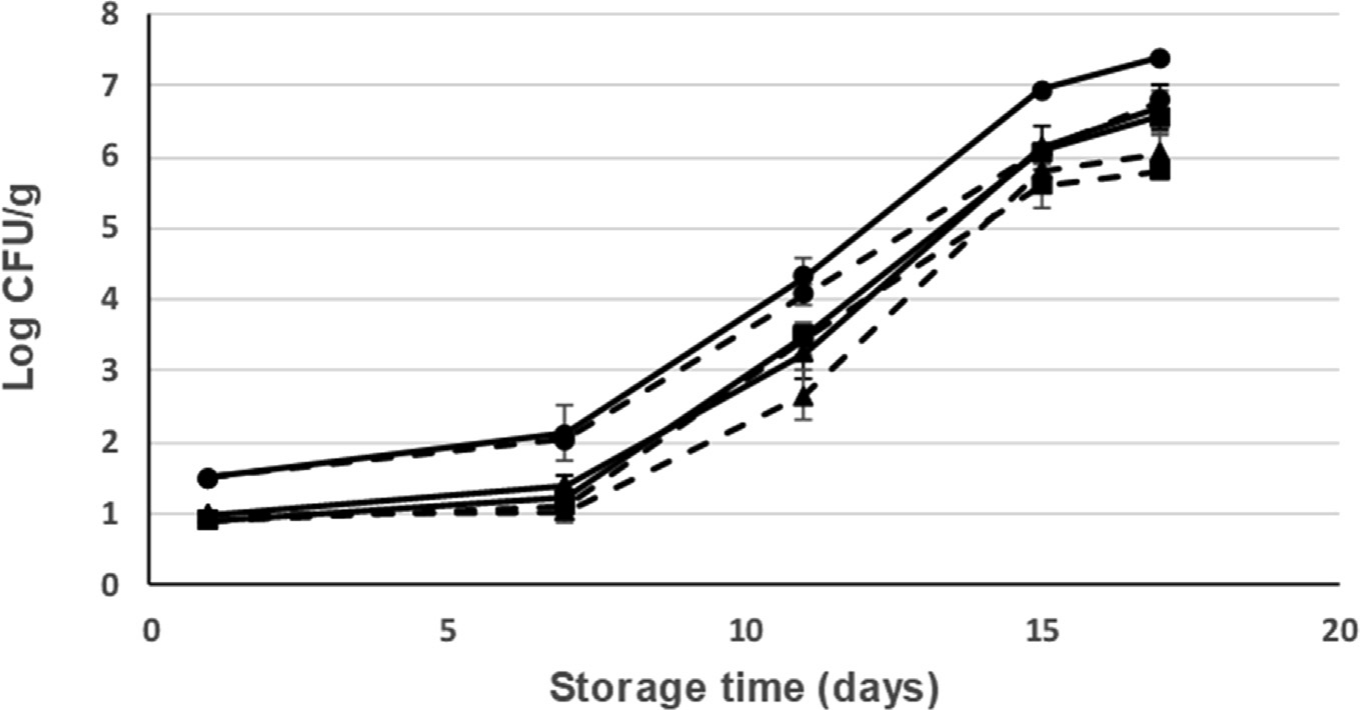
Changes in the counts of psychrophilic bacteria along cold storage (at 2 °C) of fresh seabream with different treatments: Current slaughtering and normal ice storage (control) (---●---); Current slaughtering and storage with CBG+β-CD ice (---●---; dashed line); Stunning/slaughtering with 10 mg CEO+β-CD ice and normal ice storage (---■---); Stunning/slaughtering with 10 mg CEO+β-CD ice and ice storage with CBG+β-CD ice (---■---; dashed line); Stunning/slaughtering with 15 mg CEO+β-CD ice and storage in normal ice (--▲--); Stunning/slaughtering with 15 mg CEO+β-CD ice and storage with CBG+β-CD ice (--▲--; dashed line).

The changes in the microbial flora of seabream from different treatments along ice storage are shown on Figs. 6, 7, 8, and 9. The low initial microbial count of all groups at the beginning of ice storage (day 1) indicates excellent fish quality (Figs. 6, 7, 8, and 9) although all the counts increased with storage time, mainly from the 7^th^ day.

*Enterobacteriaceae* counts showed a very low initial population indicating good fishing practises and handling. In addition, this low value indicates good hygiene of the marine environment where seabream was farmed and caught. In fact, the contribution of *Enterobacteriaceae* to the microflora of fish and its potential spoilage must be taken into consideration especially in the case of polluted water or delay in chilling after catch (Carrascosa et al., 2015). The initial *Enterobacteriaceae* counts (log CFU/g) ranged from below 1 in the different treatments. All these counts increased with ice storage time and from day 7 of ice storage. Counts in seabream stunned/slaughtered with CEO+β-CD crushed ice and ice stored with CBG+β-CD crushed ice were below counts of seabream control (4.5 log CFU/g). From the results shown in Fig. 6, it can also see that seabream stunned/slaughtered with CEO+β-CD ice decreased the final count to 3.9 log CFU/g as compared with 4.5 log CFU/g for the control. Moreover, seabream with current slaughtering and ice stored with CBG+β-CD crushed ice showed lower counts during storage time (Fig. 6).

*Pseudomonas* has been reported to be the specific spoilage bacteria in fresh Mediterranean fish stored in ice (Gennari et al., 1999). The initial *Pseudomonas* count (day 1) was lower than the detection limit in all treatments (Fig. 7). By day 11 of storage at 2 °C, *Pseudomonas* count of seabream slaughtered and ice stored with normal ice was higher than in seabream stunned/slaughtered or ice stored with ice including EOs+β-CD inclusion complex (Fig. 7). At the end of storage time (17 days), seabream control reached a count of 3.25 log CFU/g while seabream stunned/slaughtered and ice stored using crushed ice including EOs+β-CD did no exceed the value of 2.3 log CFU/g. Thus, *Pseudomonas* count was reduced by 1 log CFU/g after 17 storage days. *Pseudomonas* level grows during ice storage of fish and has been used as spoilage indicator of fresh fish due to their short generation time (Stamatis and Arkoudelos, 2007).

The initial mesophilic aerobic bacteria (MAB) low count in all sample groups (between 1 and 2 log CFU/g) indicates good fish quality (Fig. 8). MAB counts in the fresh fish are a useful tool for quality and shelf-life evaluation. During ice storage time, MAB counts in all sampling groups increased but did not exceeded the value of 7 log cfu/g considered as the maximum level for acceptability for marine fish by the International Commission on Microbiological Specifications for Foods (ICMSF, 1986; Ojagh et al., 2010) except in the case of seabream control. Seabream stunned/slaughtered with CEO+β-CD ice decreased the MAB counts to 6.5 log CFU/g as compared to 7.2 log CFU/g for the control at the end of storage time (17 days). Seabream shelf-life was determined using a regression analysis for each treatment. So, it established a microbial shelf-life of 18 days for seabream control while seabream slaughtered with ice control and ice stored in CBG+β-CD crushed ice had further shelf-life (24 days). Seabream ice stored in EOs+β-CD ice increased the shelf-life up to 4 days. In addition, seabream stunned/slaughtered with CEO+β-CD ice slurry increased the shelf-life between 2 and 3 days, with ice storage using normal ice.

The low initial psychrophilic bacteria count indicates good fish quality (Fig. 9). Psychrophilic bacteria counts in seabream control exceeded the value of 7 log CFU/g (considered as the upper acceptability limit for marine species) on 15th storage day at 2 °C. However, psychrophilic bacteria counts of seabream samples ice stored with CBG+β-CD crushed ice did not exceed 6 log CFU/g on day 17 (Fig. 9).

Psychrophilic bacteria are the major group of microorganisms responsible for aerobic spoilage of chilled stored fish (Ojagh et al., 2010). In the study of Tavakoli et al. (2018) on ice storage of whole trout, the results were similar. Control group using normal crushed ice showed the highest value of psychrophilic counts, while samples stored in ice prepared with EOs had the lowest counts. The difference between the study of Tavakoli et al. (2018) and our study is that dose of EOs used in our work is much lower (1500 mg/kg vs 50 mg/kg of storing ice).

According to Carrascosa et al. (2015), the counts of the psychrophilic microorganisms gives better results to the shelf-life estimation of chilled fish than mesophilic bacteria and 6 log CFU/g could be accepted as the acceptability limit (Mol et al., 2007). After these considerations the shelf-life of seabream control will be of 13 days, while the shelf-life of seabream stunned/slaughtered with CEO+β-CD ice slurry and ice stored with CBG+β-CD crushed ice is of 17 days. In any case, it is highlighted that seabream stunned/slaughtered using CEO-β-CD ice slurry has a greater shelf-life than seabream slaughtered with current method.

### 3.3 Physical-chemical analyses

The pH values of the fish muscle varies during post-mortem storage according to different parameters as species, season and others factors. The initial pH of seabream was between 6.35 and 6.50 and these values increased during storage at 2 °C (Table 1). The increase of pH values during storage time may be due to production by fish spoilage bacteria of basic compounds, such as ammonia compounds and TMA (Özyurt et al., 2012). There were no significant changes of pH during storage at 2 °C, and no significant differences were found across all treatment groups. The obtained pH values were similar to those previously reported by literature for seabream, when kept under storage with slurry and crushed ice at 4 °C (Kilinc et al., 2007), and stored in ice at 5 °C (Cakli et al., 2007), at the beginning of storage period. However, at the end of ice storage the observed pH values were lower than obtained by those authors. All the pH values were lower to 7.1 which is indicative of decomposition (Kilinc et al., 2007).

**Table 1:** pH changes of seabream during cold storage at 2 °C.

Parameters	Slaughter	Ice	Days				
			1	7	11	15	17
pH	Control	Control	6.50 ± 0.02^a^	6.46 ± 0.14^a^	6.25 ± 0.26^a^	6.54 ± 0.01^a^	6.65 ± 0.18^a^
		CBG-β-CD	6.43 ± 0.08^a^	6.45 ± 0.01^a^	6.40 ± 0.12^a,b^	6.46 ± 0.05^a^	6.52 ± 0.02^a^
	10 mgCEO-β-CD/kg	Control	6.47 ± 0.05^a^	6.40 ± 0.05^a^	6.57 ± 0.22^a,b^	6.49 ± 0.03^a^	6.54 ± 0.05^a^
		CBG-β-CD	6.34 ± 0.04^a^	6.23 ± 0.27^a^	6.69 ± 0.05^b^	6.50 ± 0.06^a^	6.59 ± 0.07^a^
	15 mgCEO-β-CD/kg	Control	6.40 ± 0.04^a^	6.39 ± 0.02^a^	6.65 ± 0.06^a,b^	6.53 ± 0.06^a^	6.54 ± 0.03^a^
		CBG-β-CD	6.42 ± 0.09^a^	6.36 ± 0.03^a^	6.61 ± 0.05^a,b^	6.43 ± 0.04^a^	6.60 ± 0.07^a^

NOTE: Data are expressed as means ± standard deviation (n = 3). Different letters within the column and row indicate significant differences (p ≤ 0.05).

TMA-N values are shown in Table 2. TMA-N is used as quality indicator for fish because TMA-N concentration is used to limit the acceptability of fish. The level of TMA-N value is 1 mg/100 g in incipient spoiled fish (FAO, 1986). At the beginning (day 1), TMA-N values were between 0.15 and 0.4 mg/100 g and its levels remained quite low during storage time. At the end of storage period of 17 days, TMA-N values increased in all condition. There were significant differences (*p*˃ 0.05) between control treatment group and seabream treated with EOs+β-CD ice (at stunning/slaughtering and ice-storage). Higher values were detected in seabream slaughtered and stored with control ice. These values were lower to those found for seabream when kept under storage with slurry and crushed ice at 4 °C (Kilinc et al., 2007), and ice stored at 5 °C (Cakli et al., 2007). Huidobro et al. (2001) also found a slow increase in TMA values during storage time in ice storage at 2 °C and concluded that TMA values was not a good freshness indicator in *Spararidae* species. Other authors were agree with them and considered TMA values reflected advanced spoilage and are not considered reliable for measuring the deterioration process (Özogul et al., 2007).

**Table 2:** TMA-N values (mg TMA-N/100) of seabream during cold storage at 2 °C.

Parameters	Slaughter	Ice	Days				
			1	7	11	15	17
TMA-N	Control	Control	0.39 ± 0.04^a^	0.42 ± 0.01^c^	0.44 ± 0.01^b^	0.45 ± 0.02^b^	0.52 ± 0.01^b^
		CBG-β-CD	0.37 ± 0.05^a^	0.32 ± 0.01^b^	0.32 ± 0.01^a^	0.35 ± 0.01^a^	0.41 ± 0.03^a^
	10 mgCEO-β-CD/kg	Control	0.30 ± 0.24^a^	0.31 ± 0.03^a,b^	0.37 ± 0.01^a,b^	0.41 ± 0.02^a^	0.37 ± 0.02^c^
		CBG-β-CD	0.30 ± 0.14^a^	0.30 ± 0.02^a,b^	0.39 ± 0.03^a,b^	0.40 ± 0.08^a^	0.41 ± 0.07^a^
	15 mgCEO-β-CD/kg	Control	0.30 ± 0.03^a^	0.29 ± 0.02^a,b^	0.43 ± 0.03^b^	0.36 ± 0.04^a^	0.34 ± 0.07^c^
		CBG-β-CD	0.15 ± 0.18^a^	0.23 ± 0.01^a^	0.34 ± 0.01^a^	0.35 ± 0.02^a^	0.43 ± 0.08^a^

NOTE: Data are expressed as means ± standard deviation (n = 3). Different letters within the column and row indicate significant differences (p ≤ 0.05).

With regard to WHC, all the treatments shown a decrease in WHC values throughout the ice storage time, and the WHC values showed no significant changes over the storage time (Table 3). Water loss is important because consumers consider too much exudate unattractive for consumption. The more water loss the more accumulated exudate. The WHC values ranged from 78 to 81 %, which is in accordance with results from other researchers (Alasalvar et al., 2001).

**Table 3:** Changes in WHC of seabream along cold storage at 2 °C.

Parameters	Slaughter	Ice	Days				
			1	7	11	15	17
WHC	Control	Control	79.61 ± 4.11^a^	76.67 ± 1.74^a^	77.74 ± 1.67^a^	79.83 ± 0.93^a,b^	79.35 ± 2.93^a^
		CBG-β-CD	79.67 ± 1.93^a^	77.89 ± 0.73^a^	78.79 ± 1.07^a^	81.61 ± 0.84^a,b^	81.08 ± 0.33^a^
	10 mgCEO-β-CD/kg	Control	78.01 ± 0.26^a^	78.17 ± 2.44^a^	79.36 ± 0.31^a^	78.81 ± 2.11^a,b^	74.71 ± 1.97^a^
		CBG-β-CD	78.55 ± 2.85^a^	73.86 ± 0.26^a^	78.21 ± 0.58^a^	84.32 ± 1.15^b^	76.75 ± 0.76^a^
	15 mgCEO-β-CD/kg	Control	80.30 ± 2.17^a^	76.04 ± 1.33^a^	77.93 ± 0.06^a^	75.78 ± 3.23^a^	75.88 ± 3.25^a^
		CBG-β-CD	79.24 ± 0.33^a^	76.92 ± 1.43^a^	79.53 ± 1.59^a^	78.07 ± 2.18^a,b^	75.81 ± 1.02^a^

NOTE: Data are expressed as means ± standard deviation (n = 3). Different letters within the column and row indicate significant differences (p ≤ 0.05).

Colour measurements during ice storage at 2 °C are shown in Table 4. Ocaño-Higuera et al. (2011) established changing colour was an important parameter to evaluate the quality of fresh fish. L* values increased at the end of storage period in all treatments. Other authors reported similar results with increasing L* values along storage time in ice (Kilinc et al., 2007; Cakli et al., 2006). There were significant differences in colour values in a^*^ and b^*^ parameters during the ice storage period.

**Table 4:** Changes in colour values of seabream along cold storage at 2 °C.

Parameters	Slaughter	Ice	Days				
			1	7	11	15	17
L*	Control	Control	81.89 ± 1.65^a^	76.24 ± 1.48^a^	83.36 ± 0.58^a^	86.42 ± 2.10^a^	80.78 ± 1.94^a^
		CBG-β-CD	83.56 ± 0.78^a^	76.59 ± 4.81^a^	84.90 ± 2.20^a^	88.05 ± 0.56^a^	86.34 ± 1.22^a^
	10 mgCEO-β-CD/kg	Control	81.42 ± 2.92^a^	74.16 ± 6.29^a^	81.68 ± 4.11^a^	88.24 ± 1.39^a^	82.80 ± 5.37^a^
		CBG-β-CD	83.39 ± 0.12^a^	80.03 ± 1.25^a^	83.48 ± 0.91^a^	84.14 ± 1.67^a^	83.05 ± 1.82^a^
	15 mgCEO-β-CD/kg	Control	80.40 ± 0.66^a^	80.92 ± 3.26^a^	83.31 ± 1.69^a^	87.87 ± 1.04^a^	84.19 ± 1.92^a^
		CBG-β-CD	82.73 ± 1.52^a^	76.63 ± 3.66^a^	84.29 ± 1.70^a^	84.30 ± 3.38^a^	85.59 ± 0.91^a^
a*	Control	Control	-0.32 ± 0.06^b,c^	-0.50 ± 0.05^b^	-0.76 ± 0.31^b,c^	-0.81 ± 0.09^c^	-0.42 ± 0.04^c^
		CBG-β-CD	-0.24 ± 0.11^c^	-0.27 ± 0.02^c^	-0.54 ± 0.15^c^	-0.80 ± 0.25^c^	-0.91 ± 0.17^a^
	10 mgCEO-β-CD/kg	Control	-0.45 ± 0.19^b,c^	-0.58 ± 0.21^b^	-0.85 ± 0.20^b,c^	-0.98 ± 0.29^c^	-0.51 ± 0.15^b,c^
		CBG-β-CD	-0.75 ± 0.16^b^	-0.57 ± 0.21^b^	-1.01 ± 0.08^a,b,c^	-0.87 ± 0.23^c^	-0.79 ± 0.16^a,b,c^
	15 mgCEO-β-CD/kg	Control	-0.76 ± 0.16^b^	-0.23 ± 0.14^c^	-1.08 ± 0.23^a,b,c^	-0.92 ± 0.28^c^	-0.81 ± 0.14^a,b^
		CBG-β-CD	-0.27 ± 0.22^c^	-0.52 ± 0.19^b^	-1.36 ± 0.06^a,b,c^	-1.17 ± 0.08^c^	-0.76 ± 0.12^a,b,c^
b*	Control	Control	3.26 ± 0.23^c,d^	3.20 ± 0.06^b,c^	3.35 ± 0.06^b^	3.51 ± 0.17^b^	4.21 ± 0.21^b^
		CBG-β-CD	2.37 ± 0.11^b^	3.08 ± 0.01^c^	3.18 ± 0.07^b^	3.35 ± 0.08^c^	4.07 ± 0.27^b^
	10 mgCEO-β-CD/kg	Control	3.92 ± 0.29^d^	3.91 ± 0.39^a,b^	3.76 ± 0.04^c^	4.07 ± 0.31^a^	3.50 ± 0.10^a^
		CBG-β-CD	2.70 ± 0.43^b,c^	3.23 ± 0.26^a^	3.41 ± 0.07^c^	3.51 ± 0.09^b^	3.98 ± 0.14^b^
	15 mgCEO-β-CD/kg	Control	2.90 ± 0.10^b,c^	3.00 ± 0.12^a^	1.05 ± 0.32^a^	3.78 ± 0.15^b^	3.35 ± 0.17^a^
		CBG-β-CD	2.34 ± 0.13^b^	3.50 ± 0.44^a^	2.97 ± 0.07^a^	3.31 ± 0.06^c^	4.37 ± 0.19^b^

NOTE: Data are expressed as means ± standard deviation (n = 3). Different letters within the column and row indicate significant differences (p ≤ 0.05).

### 3.4 Sensory analysis

**Fig. 10:**
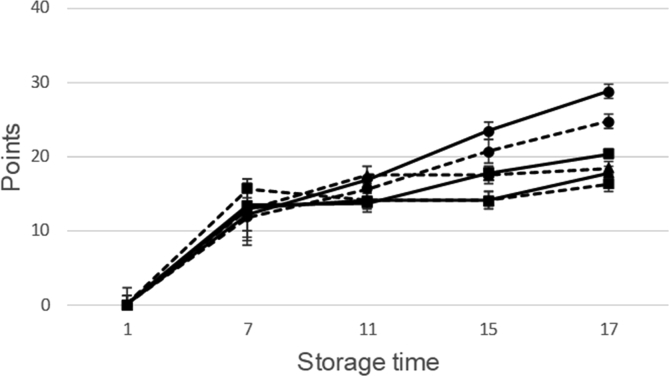
Sensory evaluation during cold storage (at 2 °C) of fresh seabream with different treatments: Current slaughtering and normal ice storage (control) (---●---); Current slaughtering and storage with CBG+β-CD ice (---●---; dashed line); Stunning/slaughtering with 10 mg CEO+β-CD ice and normal ice storage (---■---); Stunning/slaughtering with 10 mg CEO+β-CD ice and ice storage with CBG+β-CD ice (---■---; dashed line); Stunning/slaughtering with 15 mg CEO+β-CD ice and storage in normal ice (--▲--); Stunning/slaughtering with 15 mg CEO+β-CD ice and storage with CBG+β-CD ice (--▲--; dashed line). The assessment of fish was conducted using the Tasmanian Food Research unit (TRFU) system modified by Cakli et al. (2007) and Erikson et al. (2019), and developed as quality index method (QIM). The modified total QI score (Points) ranged from 0 (very fresh fish) to 40 (spoiled fish).

The panellists evaluated changes in sensory scores of seabream during ice storage (Fig. 10). Zero points show the fresher state of the fish while 40 points indicate that the fish is completely spoiled.

At the beginning of the storage period (day 1), panellists found fresh odour and typical attributes of the raw seabream in all conditions, indicating the maximum quality and freshness of seabream. Differences in sensory characteristic were found on 15th day. On day 15 seabream control (slaughtered and ice stored with normal ice) presented worst attributes: skin was dull, eyes concave and opaque cornea, gills were brown and with spoiled fish odour. Thus, the estimate shelf-life for the seabream control could be 13 days. However, on day 17 seabream stunned/slaughtered and ice stored using EOs+β-CD crushed ice was sensory acceptable but at limit of acceptability. In this way it is shown that, in this case, the count of psychrophilic microorganisms is more useful in estimating the shelf-life of the fish than MAB counts.

According to the panellists, EOs odours were not detected during ice storage. Thus, panellists did not detect differences between samples stored in control ice and samples stored with EOs+β-CD ice. Hence, EOs+β-CD ice (used with low doses in stunning/slaughtering and ice storage) had not any adverse effect on the sensory acceptability of seabream.

Similar results were found in cooked seabream (data no shown). Control seabream were worst evaluated along cold storage by the panellists. Neither odour nor taste of the cooked seabream slaughtered and stored with EOs-β-CD ice was negatively affected. The panellists did not detect unpleasant odours and taste due to EOs aroma. Thus, the use of EOs-β-CD ice in stunning/slaughtering and ice storage do not affect sensorial characteristics of fresh and cooked seabream, when low doses are used, as the handled in this work.

Our previous work showed that using 10 and 15 mg CEO+β-CD crushed ice during stunning/slaughtering promoted a decrease in plasmatic glucose levels, which confirm CEO+β-CD ice decreases the stress in fish at slaughtering (López-Cánovas et al., 2019). Furthermore, the results of the current work showed that application of CEO+β-CD crushed ice during stunning/slaughtering with or without using CBG+β-CD crushed ice during ice storage, improved the quality and freshness of seabream and extended its shelf-life. The application of EOs+β-CD improved microbiological and some chemical parameters along the storage time.

Seabream slaughtered or stored in ice including encapsulated EOs showed lower microbiological and chemical values due to the antimicrobial and antioxidant properties of the essential oils. In fact, the microbiological shelf-life was established in 18 days for seabream from control treatment while seabream slaughtered or stored in antimicrobial ice had an extended shelf-life (up to 6 days). Use of antimicrobial ice extended the shelf-life of seabream stored at 2 °C between 4 and 6 days if comparing with seabream from control treatment.

According to the results of sensory analyses, up to 15 days all the conditions were determined as fresh fish but on day 17 control seabream was no longer acceptable for consumption. Antimicrobial CBG+β-CD crushed ice avoids unpleasant sensorial attributes and improve sensorial acceptability. Thus, the application of CEO+β-CD during stunning/slaughtering, in combination with ice storing of seabream did not show off-flavour and off-odour along storage time. Thus, antimicrobial ice including CBG+β-CD can be a good option to extend shelf-life for marine species by fishing industry due to beneficial effects and the low price of EOs+β-CD crushed ice.

## Declarations

### Author contribution statement

Laura Navarro-Segura, Amanda E López Cánovas, Isabel Cabas: Performed the experiments; Analyzed and interpreted the data.

María Ros-Chumillas: Performed the experiments; Analyzed and interpreted the data; Wrote the paper.

Garcia Ayala Alfonsa: Analyzed and interpreted the data; Wrote the paper.

Antonio Lopez Gomez: Conceived and designed the experiments; Analyzed and interpreted the data; Wrote the paper.

### Funding statement

This work was funded by PESCAMUR SL Company (San Pedro del Pinatar, Murcia, Spain) and CDTI (Madrid, Spain), Project Number: IDI-20150100, and co-financed by the European Regional Development Fund (ERDF) through the Pluriregional Operational Programme for Intelligent Growth.

### Competing interest statement

The authors declare no conflict of interest.

### Additional information

No additional information is available for this paper.
